# Proposal for a Two-Tier Re-classification of Stage IV/M1 domain of Renal Cell Carcinoma into M1 (“Oligometastatic”) and M2 (“Polymetastatic”) subdomains: Analysis of the Registry for Metastatic Renal Cell Carcinoma (REMARCC)

**DOI:** 10.3389/fonc.2023.1113246

**Published:** 2023-03-29

**Authors:** Margaret F. Meagher, Maria C. Mir, Andrea Minervini, Maximilian Kriegmair, Matthias Heck, Francesco Porpiglia, Siska Van Bruwaene, Estefania Linares, Vital Hevia, Maurizio D’Anna, Alessandro Veccia, Eduard Roussel, Francesco Claps, Carlotta Palumbo, Michele Marchioni, Jonathan Afari, Cesare Saitta, Franklin Liu, Jose Rubio, Riccardo Campi, Andrea Mari, Thomas Amiel, Enrico Checcucci, Mireia Musquera, Georgi Guruli, Nicola Pavan, Maarten Albersen, Alessandro Antonelli, Tobias Klatte, Riccardo Autorino, Rana R. McKay, Ithaar H. Derweesh

**Affiliations:** ^1^ Department of Urology, University of California (UC) San Diego School of Medicine, La Jolla, CA, United States; ^2^ Department of Urology, Fundacion Instituto Valenciano Oncologia, Valencia, Spain; ^3^ Department of Urology, University of Florence, Careggi Hospital, Florence, Italy; ^4^ Department of Urology, University Medical Centre Mannheim, Mannheim, Germany; ^5^ Department of Urology, Technical University of Munich, Munich, Germany; ^6^ Department of Urology, University of Turin-San Luigi Gonzaga Hospital, Orbassano, Italy; ^7^ Department of Urology, AZ Groeninge, Kortrijk, Belgium; ^8^ Department of Urology, Hospital 12 de Octubre, Madrid, Spain; ^9^ Department of Urology, Hospital Ramon y Cajal, Madrid, Spain; ^10^ Department of Urology, Hospital Clinic, Carrer de Villarroel, Barcelona, Spain; ^11^ Department of Urology, Virginia Commonwealth University (VCU) Medical Center, Richmond, VA, United States; ^12^ Department of Urology, UK Leuven, Leuven, Belgium; ^13^ Department of Urology, University of Trieste, Trieste, Italy; ^14^ Department of Urology, Spedali Civili Hospital, University of Brescia, Brescia, Italy; ^15^ Department of Urology, University “G. d’Annunzio” Chieti-Pescara, Chieti, Italy; ^16^ Department of Urology, Royal Bournemouth Hospital, Bournemouth, United Kingdom

**Keywords:** carcinoma, renal cell, neoplasm metastasis, neoplasm staging, nephrectomy, survival analysis, TNM staging system

## Abstract

**Purpose:**

We hypothesized that two-tier re-classification of the “M” (metastasis) domain of the Tumor-Node-Metastasis (TNM) staging of Renal Cell Carcinoma (RCC) may improve staging accuracy than the current monolithic classification, as advancements in the understanding of tumor biology have led to increased recognition of the heterogeneous potential of metastatic RCC (mRCC).

**Methods:**

Multicenter retrospective analysis of patients from the REMARCC (REgistry of MetAstatic RCC) database. Patients were stratified by number of metastases into two groups, M1 (≤3, “Oligometastatic”) and M2 (>3, “Polymetastatic”). Primary outcome was overall survival (OS). Secondary outcomes were cancer-specific survival (CSS). Cox-regression and Kaplan-Meier (KMA) analysis were utilized for outcomes, and receiver operating characteristic analysis (ROC) was utilized to assess diagnostic accuracy compared to current “M” staging.

**Results:**

429 patients were stratified into proposed M1 and M2 groups (M1 = 286/M2 = 143; median follow-up 19.2 months). Cox-regression revealed M2 classification as an independent risk factor for worsened all-cause mortality (HR=1.67, p=0.001) and cancer-specific mortality (HR=1.74, p<0.001). Comparing M1-oligometastatic vs. M2-polymetastatic groups, KMA revealed significantly higher 5-year OS (36% vs. 21%, p<0.001) and 5-year CSS (39% vs. 17%, p<0.001). ROC analyses comparing OS and CSS, for M1/M2 reclassification versus unitary M designation currently in use demonstrated improved c-index for OS (M1/M2 0.635 vs. unitary M 0.500) and CSS (M1/M2 0.627 vs. unitary M 0.500).

**Conclusion:**

Subclassification of Stage “M” domain of mRCC into two clinical substage categories based on metastatic burden corresponds to distinctive tumor groups whose oncological potential varies significantly and result in improved predictive capability compared to current staging.

## 1 Introduction

Renal Cell Carcinoma (RCC) has been characterized by a stage migration over the last few decades as increasing proportion of patients are diagnosed with small and asymptomatic masses (1). Nonetheless 15-25% of patients diagnosed with RCC continue to present with metastatic disease, and up to 30%-35% of patients with localized disease develop recurrent metastatic disease (2). As understanding of both the heterogenous biological potential of renal neoplasms and patterns of progression has grown, revisions to the T3 and T4 of the Tumor-Node-Metastasis (TNM)/American Joint Committee on Cancer (AJCC) staging system have been successfully proposed and have correlated with improved prognostication and risk stratification (3).

Stage IV/metastatic renal cell carcinoma (mRCC) has been historically associated with a poor prognosis. Nonetheless, outcomes in metastatic disease are not uniform and are influenced by both patient and disease factors (4, 5). Existing risk stratification systems including Memorial Sloan Kettering Cancer Center (MSKCC)/Motzer criteria and International Metastatic RCC Database Consortium (IMDC)/Heng criteria staging have sought to stratify metastatic heterogeneity by incorporating clinical and laboratory data into prognostic groupings (6–8). Despite repeated validation studies and the instrumental role these prognostic indices have played in driving investigation and refining management recommendations for metastatic disease, the TNM/AJCC Staging for mRCC has remained constant. We sought to evaluate the impact of tumor burden on survival outcomes and hypothesized that subdividing Stage IV RCC into groups based on metastatic burden would rationalize metastatic staging and facilitate more accurate and individualized discussions with patients regarding prognosis.

## 2 Methods

### 2.1 Patient population

Our study is a retrospective international multi-institutional analysis utilizing the REMARCC (REgistry of MetAstatic RCC) database of patients presenting with metastatic RCC (mRCC) between 1/2006-10/2019 who underwent cytoreductive nephrectomy. Our patient population and methods have been described previously (9). All participating institutions received institutional review board approval. Patients referred with metastatic RCC underwent initial staging evaluation with CT or MRI of chest, abdomen, and pelvis with additional studies as indicated (10, 11). Determination of presence/extent of metastatic disease was made by treating clinicians based imaging and/or histologic findings at each participating institution (12, 13). Treatment decisions regarding cytoreductive nephrectomy, systemic therapy, and metastatectomy were conducted *via* interdisciplinary discussions between medical and urologic oncologists (14). Radiographic follow up and determination of response were conducted by RECIST (Response Evaluation Criteria in Solid Tumors) (15, 16). Patients who received cytokine or mammilian target of rapamycin (mTOR) therapy as initial therapy, and patients with non-cortical renal malignancy were excluded from analysis.

### 2.2 Data collection

Data were entered into institutional datasets by database managers. Collected variables include demographic data at time of diagnosis [age, sex, body mass index (BMI, Kg/M^2^)], baseline laboratory values [hemoglobin, lactate dehydrogenase (LDH), calcium] and clinical disease characteristics {Eastern Cooperative Oncology Group (ECOG) performance status, Karnofsky performance status, TNM stage (3), Motzer Risk Category [time from diagnosis to systemic treatment, hemoglobin concentration below lower limit of normal (3.5g/dL for men; 12.0 g/dL for women), calcium >10mg/dL, LDH >1.5 times upper limit of normal (140 U/L), and Karnofsky performance status <80%] (6), number and location of metastases}. Treatment data (cytoreductive nephrectomy, metastatectomy, systemic therapy) were collected. Survival outcomes, including progression, disease-free survival, and overall survival at last follow-up were recorded.

### 2.3 Data analysis

We *a priori* categorized Oligometastatic disease as ≤3 metastatic sites, while Polymetastatic disease was defined as >3 metastatic sites (17).We hypothesis tested this definition by performing serial Receiver Operating Characteristic (ROC)/area under the curve (AUC) analyses to analyze for C-index to determine optimal cut off point of metastatic burden to be most predictive of survival outcomes overall survival (OS), cancer-specific survival (CSS) and progression-free survival (PFS), and compared different cut offs of number of metastases (1 vs. >1, 2≤ vs. >2, ≤3 vs. >3, ≤4 vs. >4, ≤ vs. >5) for proposed M1 and M2 groups to the current M+ unitary group of the TNM/AJCC 8^th^ Edition (3).

Primary outcome was all-cause mortality (ACM)/overall survival (OS) measured from date of diagnosis to date of last follow-up or death. Secondary outcomes were cancer-specific mortality (CSM)/cancer-specific survival (CSS) and progression-free survival (PFS, defined as time to radiographic progression as per RECIST criteria) (16). The cohort was divided into M1 (oligometastatic; ≤3 metastases) vs. M2 (polymetastatic; >3 metastases) groups for descriptive analyses of demographics, clinical disease characteristics, and survival outcomes.

Descriptive analyses were conducted utilizing Student’s t-test and Fisher’s exact test for continuous and categorical variables, respectively. Cox proportional hazards regression multivariable analysis was employed for analysis of ACM and CSM, and logistic regression multivariable analysis was utilized to analyze for factors associated with progression. Kaplan Meier Analysis (KMA) was performed to analyze OS, CSS, and PFS for proposed M1 and M2 groups and M+ unitary group of the TNM/AJCC 8^th^ Edition (3). SPSS v.27 (IBM, Chicago, USA) was utilized for statistical analyses with p<0.05 considered significant.

## 3 Results

429 patients were analyzed (286 oligometastatic M1, 143 polymetastatic M2). Median follow-up for the overall cohort was 19.2 months (IQR: 6.82-38.4).

**Figure 1:**
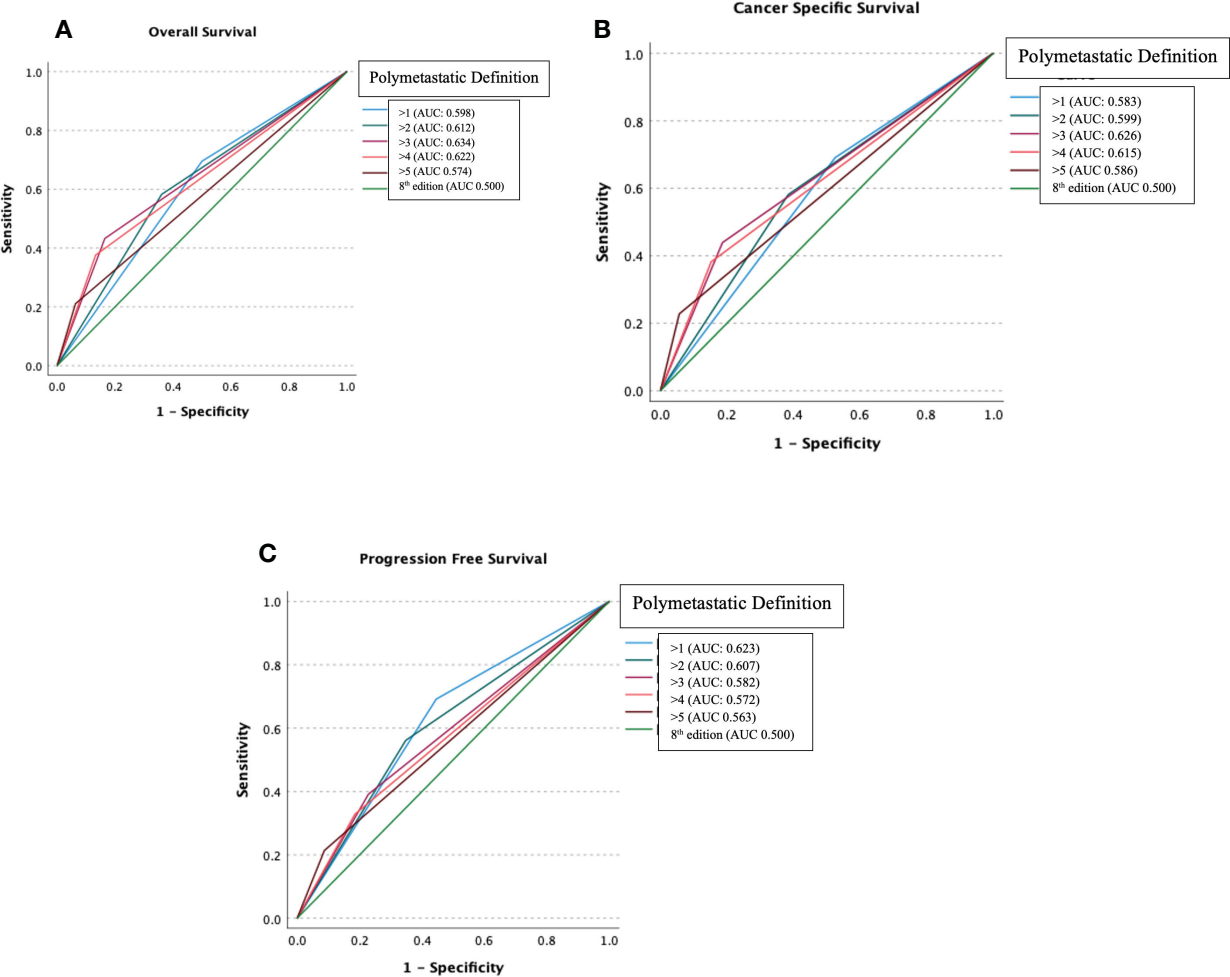
Receiver operating characteristic analysis comparing Overall Survival **(A)**, Cancer-Specific Survival **(B)** and Progression-Free Survival **(C)** for different numeric cut offs for the proposed M1/M2 reclassification (>1, >2, >3, >4, >5 metastases) versus the unitary M designation.

**Figure 1** demonstrates hypothesis testing for M1/M2 cut off of ROC analyses comparing OS, CSS, and PFS for different numeric cut offs for the proposed M1/M2 reclassification (>1, >2, >3, >4, >5 metastases) versus the unitary M designation currently in use. AUC for OS was most improved for proposed M1/M2 >3 0.634 vs. unitary M 0.500. AUC for CSS was most improved for M1/M2 >3 0.626 vs. unitary M 0.500. AUC for PFS was most improved for proposed M1/M2 >1 0.623 vs. unitary M 0.500. Based on these findings, cut off of >3 was utilized as the threshold for M1/M2 subgroups.

**Table 1** demonstrates demographic variables and clinical disease characteristics. No differences were noted between groups with respect to age (p=0.454), sex (p=0.577), BMI (p=0.192), baseline LDH (p=0.580), baseline hemoglobin (p=0.926), and clinical stage of primary tumor (p=0.160), and ECOG (p=0.334). On the other hand, patients in M1 group had lower median number of metastases at diagnosis (M1 2.0 vs. M2 6.0, p<0.001), mean calcium (M1 5.2 vs. M2 6.5, p=0.003) and patients with high Motzer risk category (M1 16.1% vs. M2 35.7%, p<0.001).

**Table 1:** Demographics, clinical disease characteristics and treatment course.

Variable	M+(n=429)	M1(n=286)	M2(n=143)	p-value
Mean Age (years, SD)	62.8 (11.0)	62.6 (11.03)	63.2 (11.02)	0.454
Sex (n, %)				0.577
Female	128 (29.8%)	88 (30.7%)	40 (28.0%)	
Male	301 (70.2%)	198 (69.3%)	103 (72.0%)	
Median BMI (kg/m^2^, IQR)	25.8 (23.1-29.0)	26.0 (23.9-29.0)	25.2 (22.5-29.0)	0.192
ECOG (n, %)				0.334
0 1 2 3	115 (36.1%) 186 (43.4%) 47 (11.0%) 41 (9.6%)	105 (36.7%) 117 (40.9%) 36 (12.6%) 27 (9.8%)	50 (34.7%) 69 (48.3%) 11 (7.7%) 13 (9.1%)	
Mean LDH (U/L ± SD)	299.5 ± 247.5	292.6 ± 287.9	310.8 ± 183.3	0.580
Mean Hgb (g/dL ± SD)	12.4 ± 2.4	12.4 ± 2.2	12.4 ± 2.2	0.926
Mean Calcium (mg/dL ±SD)	5.6 ± 5.4	5.2 ± 3.9	6.5 ± 3.6	*0.003*
Primary tumor Clinical T stage				0.160
T1	58 (13.5%)	41 (14.3%)	17 (11.9%)	
T2	95 (22.1%)	61 (21.3%)	34 (23.8%)	
T3a	128 (29.8%)	90 (31.5%)	38 (26.6%)	
T3b	80 (18.6%)	49 (17.1%)	31 (21.7%)	
T3c	9 (2.1%)	7 (2.4%)	2 (1.4%)	
T4	44 (10.3%)	23 (8.0%)	21 (14.7%)	
Clinical N stage				0.021
N0	187 (43.6%)	123 (43.0%)	64 (44.9%)	
N1	199 (46.4%)	124 (43.4%)	75 (52.4%)	
NX	32 (7.5%)	28 (9.8%)	4 (2.8%)	
Metastases at diagnosis (median, IQR)	3.5 (1.0-6.0)	2.0 (1.0-2.5)	6.0 (5.0-10.0)	<0.001
Location of Metastases at Diagnosis (n, %)				
Lungs	276 (64.3%)	173 (60.5%)	103 (72.0%)	*0.019*
Liver	52 (12.1%)	29 (10.1%)	23 (16.1%)	0.085
Bone	130 (30.3%)	94 (32.9%)	36 (25.2%)	0.119
Brain	21 (4.9%)	17 (5.9%)	4 (2.8%)	0.234
Motzer Category (n, %)				<0.001
Favorable-Risk	59 (13.8%)	47 (16.4%)	12 (8.4%)	
Intermediate-Risk	273 (63.6%)	193 (64.5%)	80 (55.9%)	
High-Risk	97 (22.6%)	46 (16.1%)	51 (35.7%)	

**Table 2** displays therapeutic interventions, survival and oncologic outcomes. M1 patients had a higher proportion of pre-surgical systemic therapy (46.5 vs. 22.4%, p<0.001), though type of therapy (p=0.153) and use of metastasectomy (p=0.121) were not different between groups. There was no significant difference in median length of follow-up for patients (M1 20.4 months vs. M2 17.5 months, p=0.230). M2 patients had a significantly greater number of progression events (69.2% vs. 53.5%, p=0.005). In addition, cancer-specific mortality events were greater in the M2 group (75.5% vs. 47.9%, p<0.001), and all-cause mortality was also greater in the M2 group (80.4% vs. 52.4%, p<0.001).

**Table 2:** Therapeutic interventions, and survival outcomes.

Variable	M+ (n=429)	M1 (n=286)	M2 (n=143)	p-value
Timing of Initiation of Systemic Therapy (n, %)				0.001
Pre CN	164 (38.2%)	132 (46.5%)	32 (22.4%)	
Post CN	234 (54.5%)	137 (47.9%)	97 (67.8%)	
Pre + Post CN	31 (7.2%)	17 (5.9%)	14 (9.8%)	
Initial Systemic Therapy (n, %)				0.153
TKI		202 (70.6%)	91 (63.6%)	
ICI		84 (29.4%)	52 (36.3%)	
Months of systemic therapy (median, IQR)	7.5 (3.0-14.1)	8.0 (2.9-14.3)	6.8 (3.0-14.5)	0.442
Initial cytoreductive Surgery				0.023
RN	372 (86.7%)	240 (83.9%)	132 (92.3%)	
PN	8 (1.9%)	6 (2.1%)	2 (1.4%)	
RN + metastatectomy	40 (9.3%)	35 (12.2%)	5 (3.5%)	
PN + metastatectomy	1 (0.2%)	0	1 (0.7%)	
RN + RT	1 (0.2%)	1 (0.3%)	0	
PN + RT	2 (0.4%)	1 (0.3%)	1 (0.7%)	
Metastatectomy Overall	104 (24.2%)	76 (26.6%)	28 (19.6%)	0.121
Metastatectomy Timing				0.057
With CN	62 (14.5%)	40 (14.0%)	22 (15.4%)	
Post-CN	14 (3.3%)	12 (4.2%)	2 (1.4%)	
Both	28 (6.5%)	24 (8.4%)	4 (2.8%)	
Median length of follow-up (months, IQR)	19.1 (6.8-38.4)	20.4 (6.3-40.5)	17.5 (8.0-34.0)	0.230
Progression (n, %)	252 (58.7%)	153 (53.5%)	99 (69.2%)	0.005
Cancer-specific mortality (n, %)	245 (57.1%)	137 (47.9%)	108 (75.5%)	<0.001
All cause mortality (n, %)	265 (61.8%)	150 (52.4%)	115 (80.4%)	<0.001

**Table 3** displays univariable and multivariable analyses for ACM, CSM, and progression. ECOG 2/3 (HR=1.79, p=0.004) and M2 polymetastatic classification (HR=1.67, p=0.001) were independent risk factors for worsened ACM, while receipt of metastatectomy (HR=0.57, p=0.005) was associated with decreased ACM. Cox regression for CSM revealed ECOG 2/3 (HR=1.98, p=0.001) and M2 polymetastatic (HR=1.74, p<0.001) to be independent risk factors for worsened CSM, while metastasectomy (HR=0.57, p=0.007) was associated with decreased CSM (**Table 3B**). Logistic regression revealed ECOG 2/3 (OR=8.35, p<0.001) and M2 polymetastatic classification (OR=2.92 p<0.001) to be independently associated with increased risk of progression, while metastasectomy (OR=0.18, p<0.001) was associated with a decreased risk of progression. Location of metastases was not significantly associated

**Table 3:** Univariable and multivariable analyses.

TABLE 3A Univariable and cox regression multivariable analysis for ACM.						
	Univariable Analysis			Multivariable Analysis		
Variable	HR	95% CI	p-value	HR	95% CI	p-value
Increasing Age (continuous)	1.01	0.99-1.02	0.280			
Sex (Male vs. Female)	1.07	0.82-1.41	0.621			
ECOG (2/3 vs. 0/1)	1.42	1.07-1.87	0.014	1.79	1.20-2.67	0.004
Increasing BMI (continuous)	0.96	0.93-0.99	0.008	0.97	0.94-1.01	0.086
Nephrectomy (yes vs. no)	0.86	0.42-1.74	0.672			
Metastasectomy (Yes vs. No)	0.61	0.45-0.82	0.001	0.57	0.39-0.84	0.005
M2 vs. M1	1.75	1.36-2.24	<0.001	1.67	1.25-2.24	0.001
Clear Cell	1.43	0.91-2.24	0.119			
Motzer score	1.58	1.27-1.96	<0.001			
Location (vs. lung referent)						
Liver	1.58	0.91-2.75	0.104			
Adrenal	0.59	0.26-1.35	0.213			
Bone	0.74	0.52-1.05	0.089			
Brain	0.80	0.35-1.82	0.601			
Other	0.65	0.35-1.21	0.654			
Multiple	1.32	0.95-1.82	0.100			
TABLE 3B Univariable and cox regression multivariable analyses for CSM.						
	Univariable Analysis			Multivariable Analysis		
Variable	HR	95% CI	p-value	HR	95% CI	p-value
Increasing Age (continuous)	1.01	0.99-1.01	0.314			
Sex (Male vs. Female)	1.16	0.87-1.55	0.317			
ECOG (2/3 vs. 0/1)	1.47	1.11-1.96	0.008	1.98	1.33-2.97	0.001
Increasing BMI (continuous)	0.95	0.92-0.98	0.007	0.97	0.94-1.01	0.080
Nephrectomy (Yes vs. No)	0.92	0.43-1.95	0.824			
Metastasectomy (Yes vs. No)	0.60	0.44-0.83	0.002	0.57	0.38-0.86	0.007
M2 vs. M1	1.77	1.37-2.28	<0.001	1.74	1.28-2.37	<0.001
Clear Cell	1.09	0.76-1.55	0.653			
Motzer score	1.59	1.26-1.99	<0.001			
Location (vs. lung referent)						
Liver	1.58	0.89-2.80	0.119			
Adrenal	0.62	0.27-1.40	0.247			
Bone	0.74	0.51-1.06	0.101			
Brain	0.84	0.37-1.90	0.670			
Other	0.64	0.33-1.22	0.172			
Multiple	1.20	0.85-1.70	0.308			
TABLE 3C Univariable and logistic regression multivariable analyses for progression.						
	Univariable Analysis			Multivariable Analysis		
Variable	OR	95% CI	p-value	OR	95% CI	p-value
Increasing Age (continuous)	0.99	0.97-1.02	0.548			
Sex (Male vs. Female)	1.12	0.67-1.88	0.668			
ECOG (2/3 vs. 0/1)	7.88	2.79-22.30	<0.001	8.35	2.89-24.13	<0.001
Increasing BMI (continuous)	0.97	0.93-1.02	0.324			
Nephrectomy (Yes vs. No)	1.03	0.72-1.48	0.870			
Metastasectomy (Yes vs. No)	0.19	0.09-0.41	<0.001	0.18	0.08-3.89	<0.001
M2 vs. M1	2.17	1.26-3.76	0.006	2.92	1.63-5.22	<0.001
Clear Cell	1.25	0.69-2.29	0.461			
Motzer score	1.36	1.06-1.74	0.016			
Location (vs. lung referent)						
Liver	0.81	0.25-2.60	0.724			
Adrenal	4.00	0.40-40.42	0.240			
Bone	0.46	0.095-2.21	0.330			
Brain	0.94	0.27-3.32	0.922			
Other	0.53	0.16-1.81	0.314			
Multiple	2.00	0.19-21.43	0.567			

**Figure 2:**
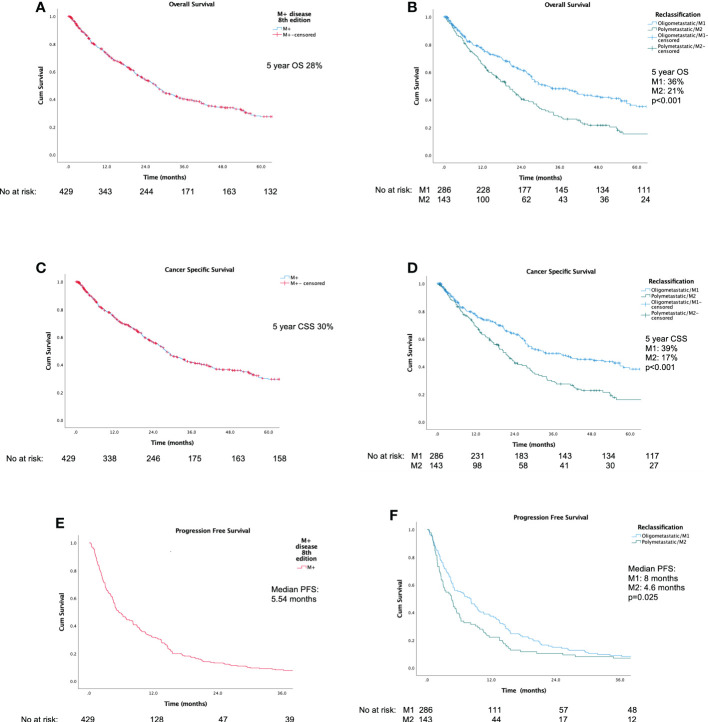
Kaplan-Meier survival analyses: **(A)** Overall Survival for current unitary M+ classification; **(B)** Overall Survival for proposed M1/M2 Subgroups; **(C)** Cancer-Specific Survival for current unitary M+ classification; **(D)** Cancer-Specific Survival for M1/M2 Subgroups; **(E)** Progression-Free Survival for current unitary M+ classification; **(F)** Progression-Free Survival for M1/M2 Subgroups.

**Figures 2A–F** display Kaplan-Meier Survival Analyses (KMA) for overall survival (OS), cancer specific survival (CSS), and progression-free survival (PFS). **Figure 2A** demonstrates KMA for OS for the entire unitary M+ cohort utilizing the current classification. 5-year OS was 28%. **Figure 2B** demonstrates KMA for OS utilizing proposed M1 and M2 subgroups. 5-year OS was significantly higher for M1-oligometastatic vs. M2-polymetastatic (36% vs. 21%, p<0.001; **Figure 2A**). **Figure 2C** demonstrates KMA for CSS for entire M+ cohort, which revealed 5-year CSS of 30%. **Figure 2D** demonstrates KMA for CSS utilizing proposed M1 and M2 subgroups. 5-year CSS was higher for M1-oligometastatic vs. M2-polymetastatic (39% vs. 17%, p<0.001) groups. **Figure 2E** demonstrated KMA for PFS of the entire M+ cohort, which revealed median PFS of 5.54 months, and **Figure 2F** demonstrates KMA for PFS utilizing proposed M1 and M2 subgroups, which demonstrated significantly longer median time to progression for M1 vs. M2 groups (8.0 months vs. 4.6 months, p=0.025).

## 4 Discussion

Utilizing an international, multi-center cohort, we present the first proposal for a reclassification of the “M” domain in TNM staging. Our findings suggest existence of two distinct subgroups driven by extent of metastatic burden, with the optimal cut off point being >3 metastases as demonstrated by ROC/AUC analyses, and is superior in its predictive ability for survival outcomes compared to the current unitary “M” domain classification. Furthermore, as evidenced in our multivariable analyses, we demonstrated that increased number of metastases was independently associated with worsened overall and cancer specific survival, which was consistent with survival estimates noted in the Kaplan-Meier analyses. While further validation is requisite, our data suggest that stratification by tumor burden may more rationally and accurately guide patient counseling and management strategy in mRCC.

Multiple studies have examined the impact of disease burden to explain heterogeneous outcomes in mRCC. In a study of 124 patients with mRCC, Iocavelli et al. found a direct relationship between tumor burden, progression-free survival, and overall survival, with each 1 cm increase in tumor burden increasing risk of progression and risk of death by 4.5% and 5%, respectively (p<0.001) (18). Number of metastatic sites has also been demonstrated to be a surrogate for disease burden (18, 19).Atzpodien studied 425 patients with mRCC and found that greater metastatic burden, defined as greater than 3 metastatic sites, was independently associated with worsened overall survival (HR=1.4, p=0.01) (20). In a previous analysis of the REMARCC database, Marchioni et al. similarly determined greater than 3 metastatic was associated with worsened overall survival (HR=1.29, p=0.040) (21). Sharma et al. analyzed 105 patients with mRCC who underwent cytoreductive nephrectomy and noted that >2 metastatic sites was independently associated with worsened OS (HR=2.09, p=0.006) (22). As demonstrated by our ROC/AUC analyses, while increasing number of metastases is indeed associated with worsened outcomes, further sub-classification of >3 distinct metastases elucidates significant differences for both overall and cancer specific survival.

The American Joint Committee on Cancer (AJCC) tumor-node-metastasis (TNM) system serves as the universally established staging method for renal cell carcinoma (23). Staging reclassifications in RCC have been proposed and adopted as understanding of tumor biology and heterogeneity has increased, with 8 editions since inception in 1977 (8, 23). Recent editions have been notable for modifications of parameters for AJCC Stage 3 and T3 RCC. In a study of 697 patients with pT3 and pT4 RCC, Thompson et al. reclassified patients into 4 prognostic groups based on direct adrenal invasion, perinephric fat invasion, and tumor thrombus level with improved prognostic accuracy (reclassification c-index 0.61 vs. 0.55) (24). Such proposals ultimately paved the way for reclassification of T3 from tumor extension into major veins, perinephric tissues, or adrenal gland in the 6^th^ edition to tumor extension into major veins or perinephric tissues but not ipsilateral adrenal gland in the 7^th^ edition (8). More recently, pT3a RCC has been further modified to include collecting system invasion in addition to renal vein/venous branches, renal sinus fat and perinephric fat between the 7^th^ and 8^th^ edition (25). To our knowledge, ours is the first attempt at reclassification of the “M” stage of the current AJCC TNM system. Our findings challenge the monolithic paradigm of “M” in TNM staging, and suggest that a reclassification of the “M” domain based on number of metastatic disease sites improves prognostic accuracy at least as well as prior modifications to Stage III disease.

Motzer (Memorial Sloan Kettering) and Heng (International Metastatic RCC Database Consortium) scores have been widely utilized and validated as prognostic schema for mRCC in contemporary cohorts (6, 7). Motzer/MSKCC scoring system is comprised of performance status, pre-operative laboratory values, and time to systemic therapy (26). Heng/IMDC criteria similarly employs performance status and time to systemic therapy, but replaces pre-operative LDH and with neutrophil and platelet couts (7). Multiple studies have sought to quantify the predictive capacity of both Heng and Motzer scores. Bamias et al. reported an AUC of 0.661 for overall survival based on Motzer in a study of 109 mRCC patients (27). Utilizing a cohort of 89 patients, Assi et al. yielded an AUC of 0.631 for overall survival for Heng criteria (28). Similarly, in a study of overall survival of 106 patients treated with sunitinib, Kwon demonstrated an AUC of 0.670 and 0.653 for Heng and Motzer, respectively (29). In a study of 628 patients with mRCC who underwent bevacizumab plus interferon treatment, Karakiewicz et al. revealed an AUC of 0.518 for progression free survival based on Motzer score (30). Analyzing Motzer criteria in our cohort, we found AUC values of 0.597, 0.588, and 0.596 for overall, cancer specific, and progression free survival, respectively. The previously demonstrated predictive capacities of Motzer and Heng scores are similar to our AUC of 0.635 for overall survival and 0.627 for cancer specific survival, as well as our AUC of 0.582 for progression free survival, suggesting that our proposed M1/M2 reclassification schema can accurately prognosticate mRCC survival outcomes in a manner similar to existing Motzer/Heng criteria. While our proposed reclassification does not aim to replace Motzer and Heng criteria, it nonetheless offers a streamlined framework within the TNM criteria in which to classify risk and counsel patients.

Our study is limited by the inherent limitations of a retrospective design. Additionally, variation in diagnostic protocols, data collection and follow-up protocols between participating centers may introduce further confounding and limitation. We acknowledge that our findings did not demonstrate location of metastasis to be associated with worsened outcomes as has been previously demonstrated in mRCC and other genitourinary malignancies which may be a result of sampling bias (31–33). Nonetheless, AUC demonstrated robust predictive ability comparable to existing risk stratification criteria for oncologic outcomes in metastatic RCC, and the diverse nature of this international registry and the robustness of the findings lend support to the validity and applicability of our findings. Our work should be regarded as hypothesis forming and as such validation by external series is requisite. Our findings suggest that subclassification of the M domain with a cut-off point of 3 metastases may optimally result into two distinct categories of outcomes based and number of metastatic sites, and presents an alternative to rationally contextualize risk, guide patient counseling, and drive further investigation.

## 5 Conclusion

Stratification of the “M” domain in mRCC into two clinical substage categories based on metastatic burden with a cut-off point of 3 metastases more accurately predicts survival outcomes, capturing lower and higher risk subsets of mRCC eclipsed by the current TNM staging, and offering a simplified alternative to more complex prognostic schemas.

## Data availability statement

The original contributions presented in the study are included in the article/supplementary materials, further inquiries can be directed to iderweesh@gmail.com.

## Ethics statement

The studies involving human participants were reviewed and approved by National Review Board. The patients/participants provided their written informed consent to participate in this study.

## Author contributions

MFM performed data collection, data analysis, and was the primary manuscript writer/editor. MCM and AMi performed data collection and manuscript writing/editing. MK, MH, FP, SB, EL, VH, MD, AV, ER, FC, CP, MMa, JA, CS, FL, JR, RC, AMa, TA, EC, MMu, GG, NP, MA, and AA were involved in data collection and data management. TK, RA, and RM contributed in protocol development and manuscript writing/editing. Finally, ID lead the team through project development and manuscript writing/editing. All authors contributed to the article and approved the submitted version.
